# Paraneoplastic Dermatoses: A Brief General Review and an Extensive Analysis of Paraneoplastic Pemphigus and Paraneoplastic Dermatomyositis

**DOI:** 10.3390/ijms21062178

**Published:** 2020-03-21

**Authors:** Dario Didona, Luca Fania, Biagio Didona, Rüdiger Eming, Michael Hertl, Giovanni Di Zenzo

**Affiliations:** 1 Department of Dermatology and Allergology, Philipps University, 35043 Marburg, Germany; ruediger.eming@med.uni-marburg.de (R.E.); michael.hertl@med.uni-marburg.de (M.H.); 2 First Dermatology Division, IDI-IRCCS, 00167 Rome, Italy; l.fania@idi.it; 3 Rare Diseases Unit, IDI-IRCCS, 00167 Rome, Italy; b.didona@idi.it; 4 Molecular and Cell Biology Laboratory, IDI-IRCCS, 00167 Rome, Italy; g.dizenzo@idi.it

**Keywords:** diagnosis, neoplasia, paraneoplastic dermatomyositis, paraneoplastic dermatoses, paraneoplastic pemphigus, skin features

## Abstract

Skin manifestations of systemic disease and malignancy are extremely polymorphous. Clinicians should be familiarized with paraneoplastic dermatoses in order to perform an early diagnosis of the underlying neoplasm. Lack of familiarity with cutaneous clues of internal malignancy may delay diagnosis and treatment of cancer. In this review, we described several paraneoplastic dermatoses and discussed extensively two paradigmatic ones, namely paraneoplastic pemphigus and paraneoplastic dermatomyositis.

## 1. Introduction

Paraneoplastic dermatoses (PD) are heterogeneous, rare, acquired diseases characterized by the presence of an underlying neoplasia. First, von Hebra in 1868 proposed the idea that sudden alterations of cutaneous pigmentation could be associated with the presence of an occult neoplasia [1,2,3]. In 1976, Curth proposed six criteria to identify a PD (Table 1) [1,3].

**Figure 1:**
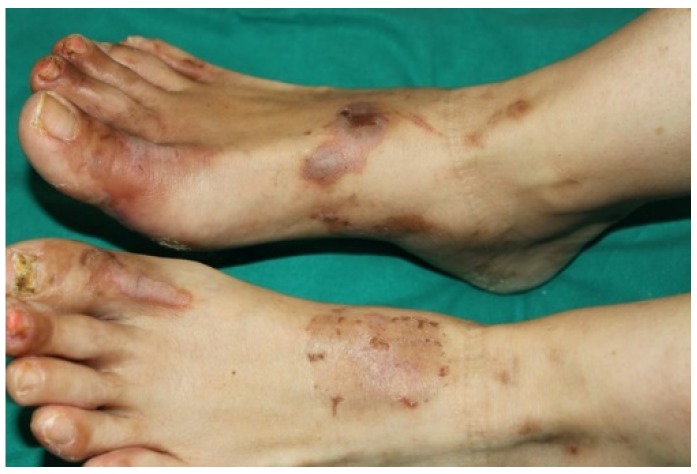
Necrolytic migratory erythema. Obligate paraneoplastic dermatosis associated with glucagonoma. Vesicles, erosions, crusts, and pustules arise at the periphery. The lesions enlarge in annular pattern, leaving pigmentation in the central area.

**Figure 2:**
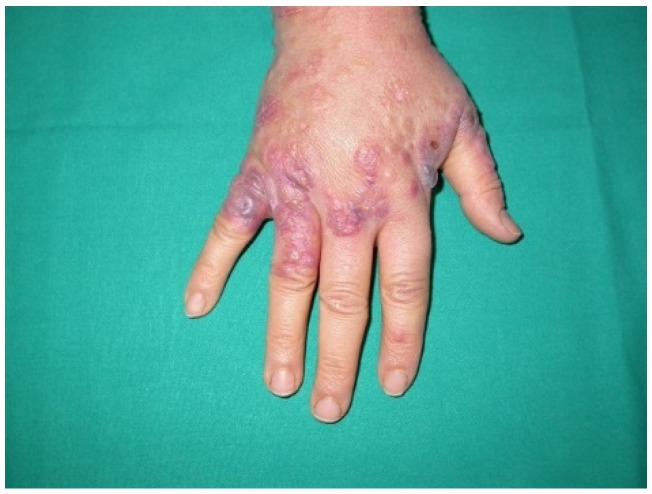
Sweet syndrome. Facultative paraneoplastic dermatosis characterized by multiple painful, sharply circumscribed dark red edematous nodules. It is usually associated with myeloproliferative and lymphoproliferative disorders.

PD usually develop simultaneously with the underlying cancer, but they can also occur before or after the development of the neoplasia [1]. Rarely, two PD can appear in the same patient [1]. The pathogenetic mechanisms of PD are still unclear. Hormones (e.g., glucagon in necrolytic migratory erythema) (Figure 1), growth factors (e.g., epiregulin in acanthosis nigricans maligna), cytokines (e.g., Sweet syndrome) (Figure 2), and antibodies (Ab) have been reported as pivotal factors in developing PD [1,3] (Table 2). Another paradigm of the PD is the absence of the neoplastic cells into cutaneous lesion [1,3]. An exception is represented by Sweet syndrome associated with a hemopoietic neoplasia, where myeloid cells are often detected in the cutaneous biopsy [4].

Based on the percentage in which a PD is associated with a neoplasm, PD are classified in two groups: Obligate PD, in which the neoplasm is present in 90%–100% of the cases, and facultative PD, in which the cancer can be detected in 25%–30% of the cases (Table 3) [1,3]. In the present review, we chose to describe extensively paraneoplastic pemphigus (PNP) for the group of obligate PD and paraneoplastic dermatomyositis (PNDM) for the group of facultative PD.

## 2. Obligate Paraneoplastic Dermatoses (PD)

### 2.1. Acanthosis Nigricans (AN)

AN is a skin condition that could be associated to obesity and insulin resistance [1,3]. AN occurs equally in both sexes without racial predilection or familial association [1,3]. The malignant form, called AN maligna (ANM), usually affects adults with an average age of 40 years [1,3]. It is mainly associated with gastrointestinal neoplasia, usually an adenocarcinoma [1,3]. ANM is characterized by a sudden onset of symmetrical hyperpigmentation of intertriginous areas, such as the axilla and the neck, although any skin area can be involved. The lesions then evolve quickly in velvety hyperkeratotic plaques, commonly surrounded by acrochordons. A generalized pruritus can be also present. An involvement of the mucosal area, including oral, anal, and genital mucosa, has been rarely described, with verrucous, flesh-colored papules. Up to 25% of the patients affected by ANM show also tripe palms (TP), also known as acquired pachydermatoglyphia, and florid cutaneous papillomatosis (FCP) [1,3]. It may also be associated with the sudden onset of multiple seborrheic keratosis (Leser–Trélat syndrome) [1,3].

Pathologically, ANM is characterized by hyperkeratosis, papillomatosis, and acanthosis [5]. The dark color is related to the hyperkeratosis. Nevertheless, an hyperpigmentation of basal layer could be observed [5].

The pathophysiological mechanism of ANM is not still completely understood. Cytokines like tumor growth factor alpha (TGF-α), insulin growth factor-like (IGF-1), fibroblast growth factor (FGF), and melanocyte-stimulating hormone (MSH) have been shown to be involved [5]. More specifically, TGF-α plays a role in keratinocytes proliferation, binding to epidermal growth factor receptor (EGFR) and leading to the activation of the mitogen-activated protein kinase (MAPK) and the extracellular-signal-regulated kinase (ERK) [5]. In ANM, EGFR has been demonstrated in basal and suprabasal keratinocytes. In addition, an increase of ERK activity has been reported in keratinocytes from ANM skin samples [5]. Furthermore, the production of IGF-1 by neoplastic cells has been reported, which could also lead to the skin changes [5].

### 2.2. Tripe Palms (TP)

Also known as acquired pachydermatoglyphia or acanthosis palmaris, TP is usually associated with Leser–Trélat sign and ANM [5]. However, some authors consider TP as a localized variant of ANM [1]. It predominantly affects adults, with a predilection for males (about 60% of cases) [1,3,5]. TP is characterized by yellowish, velvety, diffuse palmar hyperkeratosis, with accentuated dermatoglyphic patterns, that resembles the intestinal mucosa. TP is usually associated to lung and gastrointestinal cancers [1,3,5]. Pathologically, acanthosis, hyperkeratosis, and perivascular deposition of mucin in the dermis is shown [5]. Physiologically, a pivotal role of EGF-α and TGF-α released by neoplastic cells has been reported [1,3,5].

### 2.3. Necrolytic Migratory Erythema (NME)

NME represents an early sign of glucagonoma, a rare endocrine tumor of pancreatic alpha cells. Glucagonoma is more common in women after 45 years of age. It is very important to recognize its manifestations in the early stages, when the pancreatic neoplasm has not yet metastasized. The initial cutaneous manifestations are angular cheilitis and erythematous-desquamative lesions in the seborrheic areas of the face and of the nail [1,3]. Therefore, these early skin features are often confused with candida infection, which often colonizes them. In addition, desquamative lesions with peeling may appear on the legs [1,3]. In later stages, painful, pruritic mucocutaneous pinkish macules with irregular edges and annular or arciform pattern appear in intertriginous areas and distal extremities [1,3]. Differential diagnoses, such as pemphigus foliaceus, zinc deficiency, psoriasis, candidiasis, and seborrheic dermatitis, should be taken into account.

The pathological features depend on the degree of involvement. Edema, irregular acanthosis with basal cell hyperplasia, moderate perivascular inflammatory infiltrate, and parakeratosis could be observed [5]. A superficial necrosis is considered an important clue to make the correct diagnosis [5].

NME can be part of the glucagonoma syndrome, characterized by NME, glucose intolerance, and hyperglucagonemia [5]. Glucagonoma syndrome is associated with a high risk of thromboembolism, reported in about 24% of patients [5]. Other common systemic manifestations are weight loss, diarrhea, steatorrhea, abdominal pain, normocytic anemia, and psychiatric symptoms. Glucagonoma can be detected by a computed tomography (CT) scan and with somatostatin receptor scintigraphy. Rarely, small-cell lung cancer, liver cancer, insulin-secreting tumors, and duodenal neoplasms have been reported in association with NME [1,5].

The pathogenesis of NME is poorly understood. It has been reported that the reduced level of zinc and amino acids caused by tumor metabolism could determine an increase in arachidonic acid production, leading to cutaneous inflammation [1,3,5]. In addition, a deficiency of niacin, a molecule necessary for epidermal growth, could play a role in the pathogenesis of NME [1,3,5].

### 2.4. Paraneoplastic Pemphigus (PNP)

PNP is a rare autoimmune blistering disease of the skin mucous membranes, which was first described by Anhalt et al. in 1990 [6]. PNP is an obligate PD usually associated with B-cell lymphomas and hematological malignancies [7]. In 2001, Nguyen et al. suggested the term “paraneoplastic autoimmune multiorgan syndrome” (PAMS) as several organs are affected and autoantibodies bind several tissues [8,9]. PNP usually affects patients aged between 45 and 70 years [7,10,11]. However, PNP can affect every age group, including children and adolescents [12]. PNP appears to affect males and females equally [7,10,11]. Nearly 84% of all PNP are found in association with hematologic neoplasms or disorders. Among these, non-Hodgkin’s lymphoma, chronic lymphocytic leukemia, Castleman’s disease, and thymoma are mostly reported, accounting respectively for 38.6%, 18.4%, 18.4%, and 5.5% of all PNP cases [7,9,11]. Usually, the neoplasm is detected before the onset of PNP, but in about 30% of cases, the skin manifestations lead to the detection of an occult neoplasia [7,11].

The pathogenesis of PNP has been not completely elucidated. However, several auto-Ab could play a pivotal role in developing PNP. Indeed, auto-Ab directed against the plakin family are typically found in PNP, including auto-Ab against the 210 kDa envoplakin, the 190 kDa periplakin, the 250 kDa and 210 kDa desmoplakins, the 500 kDa plectin, and the 230 kDa bullous pemphigoid (BP) antigen [13,14,15]. Furthermore, auto-Ab against plakophilin 3 and desmocollin (Dsc) 1 and Dsc3 have also been detected in some studies [16,17]. In addition, auto-Ab against desmoglein (Dsg) 1 and Dsg3 may also have pathogenic activity [18,19]. Recently, the protease inhibitor α2- macroglobulin-like-1 (A2ML1) has been considered as pathogenic in PNP [20,21].

On the other hand, the cell-mediated immunity could play a role in PNP [22,23]. Indeed, the presence of selective epidermal activated CD8+ T-cells in PNP has been reported [24]. Furthermore, four PNP patients without any detectable auto-Ab have been described [25]. In addition, it has been shown that MHC-restricted CD8+ cytotoxic T-cells, non-MHC-restricted CD56+, and CD68+ natural killer (NK) cells are located at the dermo-epidermal junction of PNP lesions [26].

**Figure 3:**
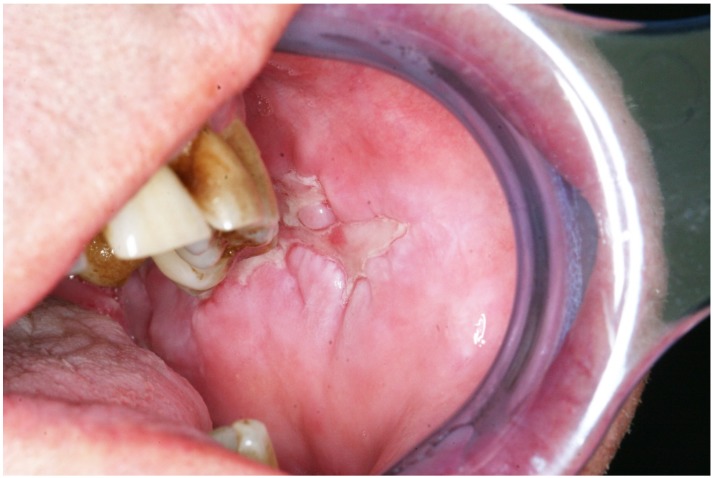
Paraneoplastic pemphigus (oral lesion). Obligate paraneoplastic dermatosis characterized by painful mouth ulcerations. It is usually associated with myeloproliferative and lymphoproliferative disorders.

**Figure 4:**
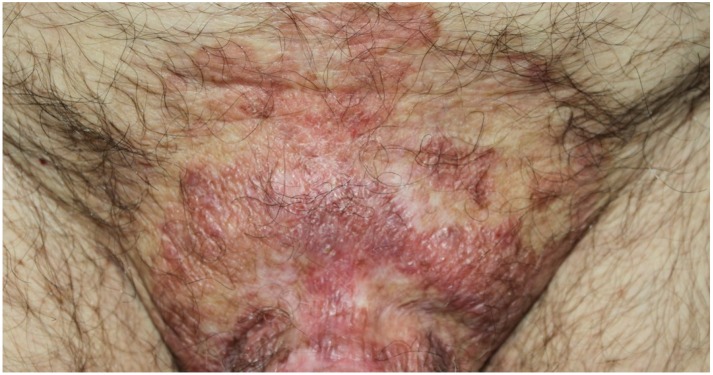
Paraneoplastic pemphigus (lichenoid lesions). Lichenoid lesions on the mons pubis. These kinds of lesions are rare in adults, but they are more often seen in children affected by paraneoplastic pemphigus.

PNP is characterized by polymorphous lesions, involving the skin and different mucosae. The variety of lesions could be explained by the different subsets of auto-Ab that could be detected in different patients [7,11]. Mucosal lesions are usually the earliest features in PNP [7,11]. Oral mucosa is always affected in PNP (Figure 3) [7,11]. Usually, severe erosions involve the vermilion of the lips and the oropharynx, causing a painful stomatitis. Mucosal lesions can also involve the nasopharynx, conjunctivae, anogenital region, and esophagus [7,11]. Cutaneous lesions usually rise after the onset of mucosal ones [7,11]. Different lesions may coexist and evolve from one type to another. Cutaneous lesions could be similar to those seen in pemphigus, pemphigoid, erythema multiforme, and graft versus host disease [7,11]. In addition, pustular and psoriasis-like lesions have been described [8]. The different clinical features could be linked to the predominance of the cell-mediated or humoral-mediated cytotoxicity [7,11,26]. Indeed, it has been reported that pemphigus lesions are prominent when the main underlying pathogenetic mechanism is the humoral-mediated cytotoxicity [7,11]. In contrast, if cell-mediated cytotoxicity is the leading pathogenetic mechanism, lichenoid lesions might be easily seen [22,25]. Lichenoid lesions are more commonly detected in children, especially on the trunk and limbs (Figure 4) [12]. PNP can also involve the respiratory epithelium in up to 92% of cases, causing dyspnea, obstructive lung disease, and bronchiolitis obliterans (BO) [7,11].

According to Anhalt et al., the diagnostic criteria include five points (Table 4) [6]. However, some different diagnostic criteria have been proposed [12,27]. Pathologically, suprabasal acantholysis with scattered inflammatory infiltrates can be detected in presence of blistering lesions, while interface and lichenoid dermatitis are more easily detected in the case of lichenoid or inflammatory skin lesions [7,11]. In direct immunofluorescence of perilesional skin or mucosa, IgG auto-Ab and/or complement deposition is observed in the epidermal intercellular spaces and/or along the basament membrane zone [28]. Circulating auto-Ab can be detected by indirect immunofluorescence (IIF) on human skin, monkey esophagus, rat bladder, and other substrates [28]. IIF on normal human skin has been found positive in up to 50% of the cases, whereas IIF on rat bladder in 75% of the cases, showing a better sensitivity [29]. Furthermore, IIF on rat bladder has shown a high specificity (83%) [29].

Radioactive immunoprecipitation is considered the gold standard in the diagnosis of PNP [30]. In addition, immunoblotting and IIF on rat bladder are useful tools for diagnosing PNP [28,30]. Immunoblotting on epidermal extracts has been used to detect 210 kDa envoplakin and 190 kDa periplakin, while immunoprecipitation can be used to identify auto-Ab against plakin family and A2ML1 [20]. By enzyme-linked immunosorbent assay (ELISA), several IgG auto-Ab can be detected, including Dsg1, Dsg3, Dsc1, Dsc2, Dsc3, and BP180 [28]. Approximately 80% of PNP patients show circulating anti-Dsg3 IgG [28]. In 2009, Probst et al. developed a new ELISA using a recombinant 56 kDa N-terminal fragment of envoplakin, which shows a sensitivity of 82% and a specificity of ≥98% [31]. More recently, a system based on nonradioactive immunoprecipitation has been reported as more sensitive than radioactive immunoprecipitation [30].

The prognosis of PNP is extremely poor, with a mortality rate of 90% [7,11]. The death is usually caused by severe complications, including sepsis, gastro-intestinal bleedings, and BO [7,11]. At this regard, a link between anti-Dsg3 antibodies and BO has been reported [32]. Therefore, it is important to evaluate accurately the respiratory symptoms in patients with positive anti-Dsg3 IgG. However, PNP cases associated with benign tumors, such as localized Castleman disease and benign thymoma, usually improve or achieve a complete remission after tumour resection [7,9].

Prednisolone in association with other immunosuppressive drugs, including azathioprine, cyclosporine, mycophenolate mofetil, and cyclophosphamide, is used as first line therapy [7,11]. In addition, the combination of prednisolone and intravenous immunoglobulins or plasmapheresis have been reported effective in selected number of patients [7,11]. Rituximab, the anti-CD20 monoclonal antibody, has been reported as effective in PNP patients with underlying B-cell lymphoma [7,11]. Whenever feasible, a complete excision of the tumor should be performed. This may cause an important improvement of the clinical features due to a dramatic reduction of autoantibodies [7,11].

## 3. Facultative Paraneoplastic Dermatoses (PD)

### 3.1. Leser-Trélat (LT)

The sign of LT has been attributed to Léser and Trélat, a German and a French surgeon, respectively, who originally associated the presence of multiple angiomas with internal malignancies [1]. LT sign has been introduced for the first time by Holländer, who described the occurrence of multiple eruptive seborrheic keratosis and underlying tumors [1]. LT sign and LT syndrome should be differentiated [3,5]. Indeed, the first expression has to be used in case of eruptive and benign multiple seborrheic keratosis, while the second one refers to lesions associated to internal malignancy [3,5]. However, the association of eruptive seborrheic keratosis and internal malignancies is controversial [3]. The hypothesis that could be a paraneoplastic syndrome is supported because it was described even in young adults [3].

LT syndrome affects individuals of both sexes equally [3]. It is characterized by verrucous, dark papules, localized mainly at the thorax and dorsum. Pruritus has been usually described as concomitant features [3,33]. LT syndrome may occur before, concomitantly, or after the diagnosis of cancer [33]. LT syndrome is mainly associated with malignancy of the gastrointestinal tract; the association of LT syndrome and lymphoproliferative malignancies is reported in about 20% of cases [3]. Pathologically, hyperkeratosis and acanthosis are described, sometimes with pseudo-horned cysts [3]. The exact pathogenesis of LT syndrome is still largely unknown, but it can be related to the secretion of EGF-α, IGF-1, and TGF-α from tumoral cells [1,5,33].

### 3.2. Pyoderma Gangrenosum (PG)

PG is a prototypic autoinflammatory neutrophilic dermatosis characterized by a spectrum of clinical presentations with variable courses. PG can manifest as painful nodules and pustules with erythematous edges and rapid evolution to deep ulcerations with undermined edges, whose debridement or surgical intervention may lead to worsening of the lesion due to pathergy [34,35,36]. Several clinical subtypes of PG have been reported, including classic ulcerative, vegetating, and bullous PG.

Classic ulcerative PG shows two distinct stages, namely the ulcerative and healing stages [36]. On the one hand, the ulcerative stage is characterized by a rapidly progressive ulceration with a peripheral red halo with raised, red-purple, undermined edges. The center of the lesion shows necrosis with a purulent or granulomatous base. Severe pain is often associated with lesion development, especially when rapid progression occurs. On the other hand, the healing stage is characterized by developing new epithelium from the edge of the wound extending into the ulcer (Gulliver’s sign). In this phase, PG heals with distinctive ‘cigarette paper-like’ or cribriform scars. Bullous PG often begins at atypical sites, such as the face, or dorsum of the hands. Bullous PG is mostly associated with underlying hematological malignancy, particularly acute myelogenous leukemia (AML). Up to 70% of cases are associated with inflammatory bowel disease and rheumatoid arthritis [34,37]. Up to 7% of PG cases are associated with an underlying neoplasia, such as myelodysplastic syndrome, myeloma, and AML [34,37]. PG can be part of several mono- or polygenetic diseases, including PAPA, PASH, PAPASH, pyoderma gangrenosum, acne conglobata, suppurativa hidradenitis, seropositive spondyloarthropathies (PASS), and psoriatic arthritis, pyoderma gangrenosum, acne, suppurativa hidradenitis (PsAPASH) syndromes [37].

Pathologically, non-specific neutrophilic infiltration in the dermis is shown [36]. Infiltration of various inflammatory cells, including histiocytes and plasma cells, and fibrosis are found in the late stage [36]. Histological features include also leukocytoclastic and lymphocyte-mediated vasculitis [36].

The pathogenesis of PG is complex. A clonality of neutrophils unrelated to underlying myeloid dyscrasia has been reported in both PG and Sweet syndrome (SS) [34,35]. In addition, the presence of clonal T-cell expansion has been reported in lesions of PG, which supports the role of aberrant T-cell response in the pathogenesis of PG [34,35]. Elevated levels of inflammatory mediators have been found in PG, suggesting a pathological inflammatory process [34,38,39]. Elevated levels of CD3+ T cells, as well as CD163+ macrophages, have been reported at wound edges of PG ulcers, while IL-8 has been found in the wound bed [34]. Therefore, T cells and macrophages have been thought to play a key role in PG disease pathogenesis. In addition, a decreased ratio of T regulatory to T helper (Th) 17 effector cells in the PG lesions [40]. Furthermore, proinflammatory cytokine expression, IL1b, and its receptor, as well as IL-8, Fas, FasL, CD40, and CD40L have been found to be significantly increased in PG lesions [38,41]. IL-23, a cytokine that plays an important role in driving IL-17-mediated and neutrophil-rich inflammation, has recently been shown to be increased in PG lesions [38].

### 3.3. Sweet Syndrome (SS)

SS is a prototypic acute febrile neutrophilic dermatosis, clinically characterized by painful, edematous, shiny erythematous nodules or plaques, which usually occur in the head, neck, and upper limbs [4]. Atypical lesions, characterized by erythematous plaques, vesicles, and bullous lesions, have also been described [4]. SS can be classified in idiopathic SS, paraneoplastic SS, and drug-induced SS. The diagnostic criteria for SS were proposed by Su and Liu and later revised by von den Driesch [3,4,5]. Pathologically, the presence of diffuse neutrophilic infiltrate in the dermis, edema, and fragmentation of the nuclei of neutrophils are described [4].

Paraneoplastic SS was first described by Cohen et al. in 1993 [4]. The clinical manifestations can precede, follow, or arise simultaneously with the diagnosis of neoplasm. Paraneoplastic SS accounts approximately for 21% of total SS cases; 85% of paraneoplastic SS are associated with hematological disorders, mostly AML [3,5]. Furthermore, paraneoplastic SS has been reported in patients affected by Hodgkin disease and polycythemia vera [3,5]. In addition, paraneoplastic SS can be associated with adenocarcinomas of the breast, genitourinary tract, and gastrointestinal tract [3,5]. Extracutaneous manifestations have been reported in circa 50% of patients affected by paraneoplastic SS [5].

In paraneoplastic SS, the over-production and dysregulation of inflammatory cytokines, like IL-1, IL-3, IL-6, IL-8, granulocyte colony stimulating factor (G-CSF), and granulocyte macrophage colony stimulating factor (GM-CSF), have been shown to be involved in the development of SS [38].

### 3.4. Paraneoplastic Dermatomyositis (PNDM)

Dermatomyositis (DM) belongs to autoimmune myositides, a group of rare autoimmune diseases, which is characterized by skin rashes and myopathy at variable degrees [42,43,44]. DM has two peaks of incidence: One in childhood between 5 and 15 years of age and one in adulthood between 40 and 60 years, with a female preponderance [42,43,44]. DM can be associated with malignancy [45]. Therefore, a screening investigation is mandatory. However, evidence-based guidelines on that topic are lacking.

The etiopathogenesis of DM is still unclear, but a range of factors, such as genetic predisposition, environment triggers, and immune- and non-immune-mediated mechanisms, play a role in the development of this disorder [43,44]. Several points support the autoimmune origin of DM. Indeed, DM may be associated to other autoimmune disorders and is characterized by several subsets of the autoantibodies. Furthermore, a DM hallmark is the presence of T-cell-mediated myocytotoxicity or complement-mediated microangiopathy [43,44]. Indeed, the primary target in DM is the endothelium of the endomysial capillaries, which is attacked by the membranolytic attack complex, formed by C3b, C3bNEO, and C4b fragments and C5b–9 [43,44]. However, specific target antigens and triggers that initiate the pathogenesis of DM have been not identified.

In the diagnosis of DM, cutaneous features play a key role. According to the criteria proposed by Bohan and Peter (Table 5), a typical skin manifestation is always necessary to diagnose DM [46,47]. In more than 50% of DM patients, skin lesions precede muscle involvement by months or years [48]. Essentially, cutaneous involvement in DM can be classified into three categories, namely pathognomonic, characteristic, and compatible clinical symptoms [49,50]. In addition, several other skin manifestations have been reported, including non-specific and rare skin features [49,50].

**Figure 5:**
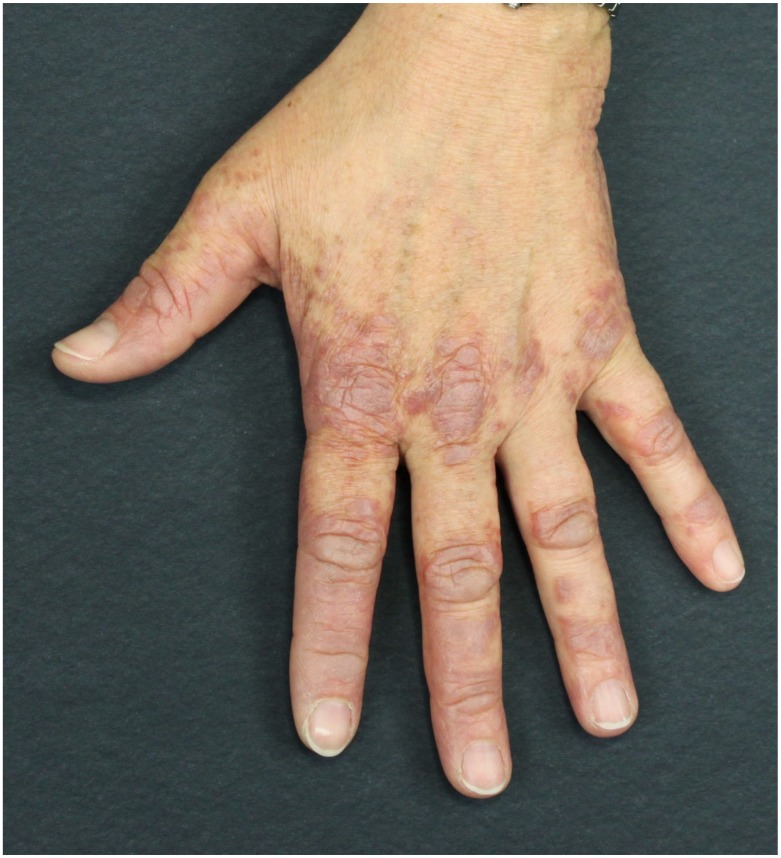
Paraneoplastic dermatomyositis (Gottron’s papules). Slightly elevated, purplish lesions on an erythematous background over bony prominences, mainly on the metacarpophalangeal, interphalangeal, and distal interphalangeal joints.

Pathognomonic skin features are Gottron’s papules and Gottron’s sign. On the one hand, Gottron’s sign is characterized by erythematous macules in a linear arrangement on the extremities, manly accentuated on the dorsal and lateral side of hands and fingers. Usually, it is associated with later desquamation. Gottron’s sign can be also detected on other body areas, mainly the knees and elbows. On the other hand, Gottron’s papules present as slightly elevated, purplish lesions on an erythematous background over bony prominences, mainly on the metacarpophalangeal, interphalangeal, and distal interphalangeal joints (Figure 5). Gottron’s papules are usually detected also on the nail borders.

**Figure 6:**
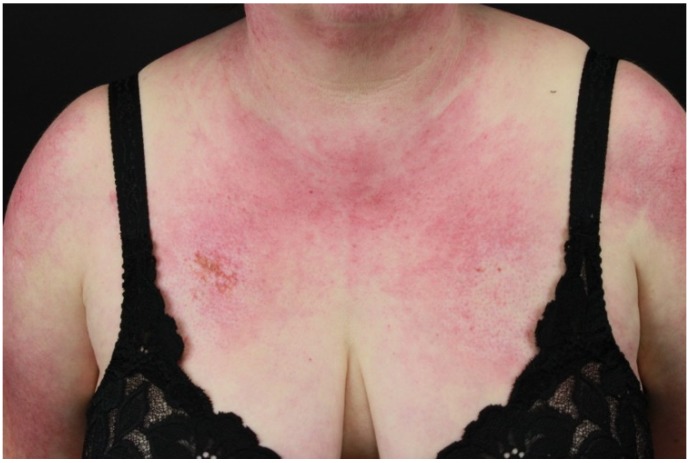
Paraneoplastic dermatomyositis (V-sign). Erythematous maculopapular rash of the V area of upper chest.

Characteristic skin features include heliotrope rash, shawl and V-sign, nail-fold changes (Keining’s sign), and scaly dermatitis of the scalp. Heliotrope rash presents as symmetric purplish erythema with edema involving mainly the upper eyelids. It is usually associated with pruritus. Heliotrope rash can also involve the cheeks, the nose, and the nasolabial folds. Occasionally, the heliotrope rash presents only as subtle mild discoloration of the eyelid borders. Shawl and V-signs are represented namely by erythematous maculopapular rash of the upper back and deltoids (shawl-sign), and V area of upper chest (V-sign) (Figure 6). Characteristic nail-fold features are represented by periungual telangiectasia with dystrophic or overgrowth cuticles, and small hemorrhagic infarcts. This phenomenon is called Keining’s sign. Scalp involvement manifests with a dusky erythematous scaly dermatitis, usually misdiagnosed as seborrheic dermatitis or psoriasis. Usually, it is associated with intense pruritus. In some patients, non-scarring alopecia has been reported, usually in association with a flare of the systemic disease [44].

Compatible skin signs are represented by poikiloderma (the combination of atrophy, dyspigmentation, and telangiectasia) on photo-exposed areas, holster sign, periorbital edema, and facial swelling. Poikiloderma usually affects the upper chest and the buttocks; it can be also detected on thighs and hips. It is usually asymmetric and has a chronic course. The holster sign is characterized by poikiloderma of hips and lateral thighs, resembling a handgun holster. Bilateral periorbital purple edema has been also described and may cause facial swelling [51].

Less commonly, cutaneous vasculitis manifestations and calcinosis cutis have been described in DM patients [52]. Cutaneous vasculitis can manifest as vesicles, necrosis, erosions, or ulcerations. In the majority of cases, cutaneous vasculitis has been reported in juvenile DM. Palpable purpura, urticarial lesions, livedo reticularis, digital, and oral ulcers have been also described in juvenile DM patients with vasculitis. Furthermore, vasculitic skin manifestations have been mainly associated with underlying malignancy [52]. Calcinosis cutis is characterized by cutaneous and/or subcutaneous calcium deposits. Clinically, it manifests as bump nodules, mainly located on elbows, knees, and buttocks. Calcinosis cutis has been frequently related to solid neoplasia or blood malignancy [53].

The association between myositis and neoplasia was firstly described in 1916, when Stertz reported a case of myositis in a patient with gastric cancer [54]. In a recent metanalysis, a prevalence of neoplasia in 14.8% of DM patients has been reported [54].

The relative risk of carcinoma in DM ranges between 3% and 8% [55,56,57]. Furthermore, a slightly more elevated relative risk has been reported in the male population [54,58]. As expected, the risk of malignancy increases with the age of the patients. Indeed, the relative risk of malignancy was 2.79 for patients <45 years and 3.13 for those >45 years [54,58]. The risk of malignancy among DM patients is higher in the first year after diagnosis (especially higher in the first three months after diagnosis of DM) [57], then steadily decreases through five years, but remains persistently slightly elevated in comparison to the general population [54,58].

Lung and gastrointestinal neoplasia have been mostly reported in DM patients [55,56,57]. In addition, plenty of cases of malignancy of nasopharynx have been described [59,60]. However, different malignancies have been reported in association with DM, including ovarian, breast, prostate, kidney cancer, and different types of hematological malignancies [55,56,57,59,60,61]. In addition, the significant variety in malignancies associated with DM may reflect differences in malignancy risk across different populations. Indeed, in a Taiwanese study, the most commonly associated malignancy was nasopharynx carcinoma [60], while in a Japanese study, gastric cancer was the most frequently detected neoplasia in DM [45]. In addition, the incidence of ovarian cancer in DM patients has been recently revaluated [54,58]. Indeed, previous papers reported a 10-fold increase in the risk of ovarian cancer in female DM patients [55,57]; however, a recent study described only a five-fold increased risk [58].

It has been hypothesized that the increased incidence of malignancy in DM patients may be partially due to a more complete cancer screening in this population [58]. However, a higher risk of malignancy has been reported in DM patients also before the onset of cutaneous or muscular features [43,44]. Indeed, cancers may be detected prior, concurrently, or after the onset of DM [43,44]. In juvenile DM, malignancies have been rarely reported; however, it is important to perform a comprehensive cancer screening in atypical cases or in patients with splenomegaly or lymphadenopathy [62,63].

In DM patients, a broad cancer screening should be performed, although no guidelines have been published [64]. However, a recent Spanish study proposed to perform chest, abdomen, and pelvic CT scan in all newly diagnosed DM patients [65]. Recently, an association between anti-TIF1 (p155/140) IgG and paraneoplastic DM has been described [66]. Indeed, it has been postulated a shared antigen expression between regenerating muscle cells and cancer cells [66].

Immunosuppressive drugs represent the mainstay of therapy for DM. A consensus about the therapy has been not yet reached because of the lack of consensus on classification, of relevant clinical trials, and of standardized outcome measures [2,3]. In planning a treatment regimen, several factors should be taken into account, such as age of the patient, disease activity, and comorbidities. Indeed, a delay in settling the therapy will result in a worse prognosis and outcome. Whenever feasible, a complete excision of the tumor should be performed.

High-dose corticosteroids (1 mg/kg/day) are considered the first-line therapy. In the case of no improvement after 3–6 months or in the case of relapses while tapering, a second-line immunosuppressive agent should be considered as combination therapy [2,3]. Second-line immunosuppressive drugs include azathioprine, methotrexate, mycophenolate mofetil, rituximab, and intravenous immunoglobulin [2,3].

## 4. Conclusions

Skin manifestations of neoplasia are protean. Their recognition can lead to a prompt cancer detection and to an early start of the appropriate therapy. PNP and PNDM are two of the most paradigmatic PD, that should be promptly recognized to start an adequate systemic therapy. Therefore, dermatologists and clinicians too should be familiarized with PD to perform an early diagnosis of the underlying neoplasm.

## Figures and Tables

**Table 1:** Curth criteria for paraneoplastic dermatoses

Criterion
1. The onset of dermatosis must be near to the beginning of the neoplasia
2. Both must follow parallel courses
3. The dermatosis must not be part of any genetic syndrome
4. A specific dermatosis accompanies a specific tumor
5. The cutaneous disease is rare in general population
6. There is a high grade of association with the neoplasia

**Table 2:** Molecular findings in paraneoplastic dermatoses.

Paraneoplastic Dermatosis	Involved Molecular Factors and Detected Antibodies
Acanthosis nigricans [1,2,3]	FGF, IGF-1, MSHα, TGF-α
Tripe palms [1,2,3]	EGF-α and TGF-α
Necrolytic migratory erythema [1,2,3]	Increased level of arachidonic acid and deficit of niacin
Paraneoplastic pemphigus [1,2,3]	Circulating autoantibodies against α-2-macroglobulin-like-1, bullous pemphigoid antigen, desmocollins 1 and 3, desmogleins 1 and 3, desmoplakins 1 and 2, envoplakin, and periplakin, and plakophilin 3
Leser-Trélat [1,2,3]	EGF-α, IGF-1, and TGF-α
Pyoderma gangrenosum [1,2,3]	Fas, FasL, IL1b, IL-8, IL-17, IL-23
Sweet syndrome [1,3,4,5]	G-CSF, GM-CSF, IL-1, IL-3, IL-6, IL-8
Paraneoplastic dermatomyositis [1,2,3]	Circulating autoantibodies against NXP-2 and TIF1-γ

Abbreviations: Epidermal growth factor alpha (EGF-α), Fas ligand (FasL), fibroblast growth factor (FGF), granulocyte colony stimulating factor (G-CSF), granulocyte macrophage colony stimulating factor (GM-CSF), insulin growth factor-like (IGF-1), interleukin (IL), melanocyte-stimulating hormone (MSH), nuclear matrix protein 2 (NXP-2), transcriptional intermediary factor 1 gamma (TIF1-γ), tumour growth factor alpha (TGF-α).

**Table 3:** Obligate and facultative paraneoplastic dermatoses (PD).

**Neoplasia**	**Obligate PD**
Gastrointestinal tract	Acanthosis nigricans maligna Necrolytic migratory erythema Leser–Trelat’s syndrome Trousseau’s syndrome
Myeloproliferative and lymphoproliferative disorders	Paraneoplastic pemphigus
Miscellaneous	Acquired hypertricosis lanuginose Acrokeratosis paraneoplastica of Bazex Erythema gyratum repens
**Neoplasia**	**Facultative PD**
Low respiratory tract and gastrointestinal tract	Paraneoplastic dermatomyositis
Esophageal tract	Acquired palmo-plantar keratoderma
Myeloproliferative and lymphoproliferative disorders	Sweet syndrome Pyoderma ganrenosum
Miscellaneous	Pytiriasis rotunda Multicentric reticulohistiocytosis Anti-laminina 332 bullous pemphigoid

**Table 4:** Diagnostic criteria for the diagnosis of paraneoplastic pemphigus (adapted from [7]).

Criterion	Details
Clinical features	Painful mucosal erosions with or without a multiform skin eruption characterized by blisters and erosions, occurring in association with an occult or evident neoplasm
Pathology	Suprabasal intraepithelial acantholysis, interface dermatitis, and necrosis of keratinocytes
Direct immunofluorescence	Combined presence of IgG and complement granular-linear deposition within the epidermal intercellular spaces and along the basement-membrane zone
Indirect immunofluorescence	Presence of circulating antibodies directed against the intercellular zone of stratified squamous or transitional epithelia
Immunoprecipitation	Complex of proteins, including desmoplakin 1 (250 kDa), bullous pemphigoid antigen (230 kDa), envoplakin (210 kDa), desmoplakin 2 (210 kDa), periplakin (190 kDa) and α-2-macroglobulin-like-1 (170 kDa)

**Table 5:** Diagnostic criteria for dermatomyositis proposed by Bohan and Peter.

Criterion	Details
1. Symmetric proximal muscle weakness	Dysphagia and/or diaphragmatic weakness can be present
2. Increase of skeletal muscle enzymes	High level of skeletal muscle enzymes, such as creatine kinase, aspartate transaminase, alanine transaminase, andlactate dehydrogenase
3. Alteration at EMG	Several abnormalities can be detected, including positive sharp waves, and repetitive high-frequency discharges
4. Alterations showed in muscle biopsy	Several pattern can be showed, including loss of capillaries, deposits of C5b–C9 on the capillaries, and endothelial microtubular inclusions
5. Typical skin rash	Heliotrope rash or Gottron’s sign

Definite dermatomyositis requires criterion nr.5 and at least 3 of criteria number (nr.) 1–4. Probable dermatomyositis requires criterion nr.5 and at least 2 of criteria nr. 1–4. Possible dermatomyositis requires criterion nr.5 and at least 1 of criterion nr. 1–4.
